# Seasonal variation of diet quality in a large middle-aged and elderly Dutch population-based cohort

**DOI:** 10.1007/s00394-019-01918-5

**Published:** 2019-02-08

**Authors:** Janine E. van der Toorn, Magda Cepeda, Jessica C. Kiefte-de Jong, Oscar H. Franco, Trudy Voortman, Josje D. Schoufour

**Affiliations:** 1 Department of Epidemiology, Erasmus MC, University Medical Centre, PO Box 2040, 3000 CA Rotterdam, The Netherlands; 2 Leiden University College, The Hague, The Netherlands

**Keywords:** Seasonality, Diet quality, Food frequency questionnaire, Food groups, Dietary guidelines

## Abstract

**Purpose:**

Several studies have reported seasonal variation in intake of food groups and certain nutrients. However, whether this could lead to a seasonal pattern of diet quality has not been addressed. We aimed to describe the seasonality of diet quality, and to examine the contribution of the food groups included in the dietary guidelines to this seasonality.

**Methods:**

Among 9701 middle-aged and elderly participants of the Rotterdam Study, a prospective population-based cohort, diet was assessed using food-frequency questionnaires (FFQ). Diet quality was measured as adherence to the Dutch dietary guidelines, and expressed in a diet quality score ranging from 0 to 14 points. The seasonality of diet quality and of the food group intake was examined using cosinor linear mixed models. Models were adjusted for sex, age, cohort, energy intake, physical activity, body mass index, comorbidities, and education.

**Results:**

Diet quality had a seasonal pattern with a winter-peak (seasonal variation = 0.10 points, December-peak) especially among participants who were men, obese and of high socio-economic level. This pattern was mostly explained by the seasonal variation in the intake of legumes (seasonal variation = 3.52 g/day, December-peak), nuts (seasonal variation = 0.78 g/day, January-peak), sugar-containing beverages (seasonal variation = 12.96 milliliters/day, June-peak), and dairy (seasonal variation = 17.52 g/day, June-peak).

**Conclusions:**

Diet quality varies seasonally with heterogeneous seasonality of food groups counteractively contributing to the seasonal pattern in diet quality. This seasonality should be considered in future research on dietary behavior. Also, season-specific recommendations and policies are required to improve diet quality throughout the year.

**Electronic supplementary material:**

The online version of this article (10.1007/s00394-019-01918-5) contains supplementary material, which is available to authorized users.

## Introduction

There are several approaches to study diet behavior, including the ‘nutrient approach’, ‘foods or food group approach’ and ‘dietary pattern approach’ [1, 2]. Studying the role of foods and specific nutrients in health has led to important findings with relevant implications [3], such as the improvement of food products based on the evidence on the adverse effects of trans-fatty acids [4]. However, the high level of inter-correlation between nutrients in the diet makes a focus on studying intake of single nutrients challenging [2]. Also, for the public it could be difficult to interpret findings on specific nutrients and to translate this into diets. Complementary to the food and nutrient approach, nutrition research is increasingly focusing on a dietary pattern approach, which captures the totality of the diet [1, 2, 5], for example, using diet quality scores.

Several factors determine the diet quality of individuals. Diet quality varies across age groups, sex, ethnicity [6], and socio-economic status (SES) [7, 8]. Emerging evidence shows that diet is not constant throughout the year, as nutrient and food groups intake follow a seasonal pattern [9–12]. Nevertheless, less is known about how diet quality varies throughout the year and how food groups interact to convey such variation.

Understanding the seasonality of diet quality can contribute to unveil determinants underlying the variation between seasons of diet behavior as a lifestyle factor and the seasonality of diet-related morbidity and mortality [13, 14]. It also contributes to the ongoing debate regarding the factors that could be efficiently targeted to improve diet quality and to identify the role of specific food groups on diet quality. Therefore, we aimed to describe the seasonality of diet quality defined as adherence to dietary guidelines, and to examine which food groups included in these guidelines explain the seasonal pattern of diet quality in the population of the Rotterdam Study.

## Materials and methods

### Study design and participants

This is a cross-sectional analysis based on the Rotterdam Study, a large prospective population-based cohort initiated in 1989 including adults living in the Ommoord district in Rotterdam, the Netherlands. The Rotterdam Study was initially designed to examine risk factors of cardiovascular, neurological, respiratory, psychiatric, locomotor, ophthalmological, endocrine, and dermatological diseases [15]. The study is composed of three sub-cohorts (RS-I: 7893 participants aged 55 years or above; RS-II: 3011 participants aged over 55 years of age or who moved into the district; and RS-III: 3932 participants aged 45 years and over). Study visits are scheduled throughout the year at participant convenience. Follow-up visits are performed every 4–5 years [15].

We selected cohort visits with available data of dietary intake using a semi-quantitative food frequency questionnaire (FFQ), i.e., first and fifth visits of first cohort (RS-I-1, RS-I-5), first and third visits of the second cohort (RS-II-1, RS-II-3), and first visit of the third cohort (RS-III-1). Each participant contributed with up to two visits (observations). Out of 13,008 observations with diet data available, we excluded those that reported an unreliable dietary intake according to the trained dietician who performed the interviews or because the daily energy intakes were implausible, for which cut-offs were set at < 500 kcal or > 5000 kcal/day (*n* = 419). Consequently, our sample was 12,589 observations obtained from 9,701 participants (full flowchart provided in Supplemental Fig. 1).

### Diet quality assessment

For visits RS-I-1 and RS-II-1, an FFQ with a two-stage approach was used. First, using a self-administrated checklist with 170 food items, participants indicated which food groups they consumed at least twice a month during the preceding year. In the second stage, participants had an interview with a trained dietician who used the 170-item checklist to identify the amounts of food intake over the past year. This FFQ was previously validated against four 24-h urinary excretion samples and fifteen 24-h dietary records, which showed adequate ability to rank participants’ food group and nutrient intake [16]. For visits RS-I-5, RS-II-3 and RS-III-1, an extended self-administrated FFQ based on 389 food items about the frequency and amount of consumed food items in days, weeks, and months according to the previous month was used, and filled out at home. This FFQ was previously validated against a 9-day dietary record and a 4-week dietary history among two Dutch populations [17, 18]. To estimate portion sizes in grams, standardized household measures were applied [19]. For calculation of the nutritional data, the Dutch Food Composition Table (NEVO) was used [20].

Based on the FFQ, adherence to the Dutch dietary guidelines was calculated and expressed in a score [21, 22]. This a priori dietary index is based on the Dutch dietary guidelines 2015 for an optimal healthy diet [22, 23], consisting of fifteen components: vegetables and fruit, whole grain products, legumes, nuts, dairy, fish, tea, coffee, unsaturated fat and oil ratio, whole grain ratio, red and processed meat, sugar-containing beverages, alcohol, salt, and supplement use [23]. For the purpose of this study, we omitted coffee and supplements because no complete information was available [22]. Adherence for each food group was predefined at specific cut-off values (Table 1); adherence per food group was scored as 1 and non-adherence as 0. Thus, total diet quality ranged from zero to fourteen points, with a higher score representing a higher adherence, i.e., a better diet quality.

**Table 1:** Components of the Dutch dietary guidelines 2015 and corresponding cut-off scores

	Food groups	Guideline
1	Vegetables	≥ 200 g/day
2	Fruit	≥ 200 g/day
3	Whole grain products	≥ 90 g/day
4	Legumes	≥ 135 g/week
5	Nuts	≥ 15 g/day
6	Dairy	≥ 350 g/day
7	Fish	≥ 100 g/week
8	Tea	≥ 450 ml/day
9	Unsaturated fat and oil ratio	Replace fats ≥ 50% of total fats as healthy fats^a^
10	Whole grain ratio	Replace refined grains ≥ 50% of total grains as whole grains
11	Red and processed meat	< 300 g/week
12	Sugar-containing beverages	< 150 ml/day
13	Alcohol	≤ 10 g/day
14	Salt	≤ 6 g/day

^a^Total fats: margarine, oils and butter. Healthy fats: soft margarine, oils

### Covariate assessment

Data collection included a standardized home interview and two visits to the research center for clinical examination and blood sampling. Energy intake was estimated from FFQ responses. Weight and height were measured with participants standing straight without wearing shoes or heavy clothes. Weight was measured in kilograms using an electronic floor scale and height was measured in centimeters with a wall-mounted stadiometer. BMI was calculated dividing weight by height squared (kg/m^2^), and was stratified into normal weight (18.5–25 kg/m^2^) and overweight/obesity (> 25 kg/m^2^). Participants’ level of education, monthly household income, living status and smoking behavior was obtained by trained interviewers. Level of education was expressed in primary (primary education), low–intermediate (lower/intermediate general education or lower vocational education), intermediate–high (intermediate vocational education or higher general education) or high (higher vocational education or university). Monthly household income was classified as <€1,500 or ≥ €1,500. Education and income information were used to calculate SES; low SES was defined as low primary/low education level or income below <€1500, high SES was defined as intermediate/high education and income ≥€1,500 [8, 24, 25]. Living status was expressed as ‘living alone’ or ‘living with partner, relatives, or others’. Smoking status was expressed as ‘never smoked’, ‘ever smoked’, or ‘current smoker’. Prevalent comorbidities was determined by a combination of blood examinations, continuous digital linkage of medical records and by information of medical specialists [26–28], it was operationalized as “yes” if at least one of the following was present: myocardial infarction (MI), stroke, type 2 diabetes mellitus (T2DM), and cancer, and “no” otherwise. Physical activity at RS-I-3 (as a proxy for RS-I-1) and RS-II-1 was assessed using a validated version of the Zutphen Physical Activity Questionnaire [29], and was expressed in MET-hours/week [30]. At RS-I-5, and RS-II-3, physical activity was assessed using the LASA Physical Activity Questionnaire (LAPAQ), and expressed in MET-hours/week [31]. We accounted for heterogeneity between the questionnaires by estimating a cohort and follow-up visit-specific z score of the MET-hours/week.

### Statistical analyses

Characteristics of the participants at study visit are described per season using descriptive statistics. Absolute values and percentages were used for categorical variables and medians and interquartile ranges (IQRs) for continuous variables; differences per season were tested with Chi-square test and Kruskal–Wallis test, respectively. Seasons were defined according to the light season definition, centered at the equinoxes (winter: November 6–February 4; spring: February 5–May 6; summer: May 7–August 5; and fall: August 6–November 5) [32].

To account for potential bias associated with missing data, we imputed missing values of covariates using multiple imputation (*n* = 5 imputations) by chained equations [33]. Further details of imputation procedures are provided in Supplemental Fig. 1.

We examined the seasonality of diet quality and daily intake (grams, milliliters or ratio per day) of each food group using cosinor linear mixed models [9]. Date of study visit was included in the model transformed into its cosinor terms (i.e., sine and cosine) [13, 34] with an assumed annual seasonality [10]. The model was further adjusted for age, sex, cohort, kilocalories/day (Model 1). The coefficients of the cosinor terms were used to calculate the amplitude, seasonal variation, and the date with the highest (peak) or lowest (nadir) diet quality score [34]. The amplitude is the distance from the annual average of diet quality to the peak or the nadir. The seasonality was presented as the seasonal variation, which is the maximal difference between the peak and nadir, i.e., 2*amplitude. Detailed descriptions to estimate the amplitude, seasonal variation, peak, and nadir are provided elsewhere [13, 34]. The variance of the seasonal variation was estimated using the delta method [35].

Model 2 was fitted to examine the seasonality of the diet quality after taking into account the non-random attendance of the participants to the study center throughout the year. The potential covariates were selected on the basis of literature [7, 36–38], of the differences of the population at specific periods of the year, and the percentage of change in the amplitude. The final set of covariates included physical activity, BMI, smoking, prevalent comorbidities, living status, income and education (Model 2).

Subsequently, we examined the seasonality of each food group included in the Dutch dietary guidelines. Model 1 and Model 2 were fitted for each of the fourteen food groups, using as outcome the continuous daily intake of each food group. The seasonality of total energy intake was also examined. To provide consistency and comparability, Models 1 and 2 included the same covariates as for the diet quality score. To examine what food groups contributed the most to the seasonality of diet quality, we re-calculated the seasonal variation of the diet quality score after excluding one food group at a time from the total score.

Finally, we performed several subgroup analyses to identify effect modification. We performed stratified analyses for age [39, 40], sex [41], BMI [42], SES [8, 24], and living status [43, 44]. As two different types of FFQs were used to measure dietary intake, we also performed a stratified analysis to assess differences in seasonality of diet quality according to FFQ. Finally, to better characterize the population according to diet quality score, we compared participants with low diet quality (below one standard deviation of adjusted average diet quality score), high diet quality (above one standard deviation), and intermediate diet quality (in between low and high diet quality). Data were analyzed using STATA v.14 (StataCorp). We followed the STROBE guidelines for reporting of cross-sectional studies (Supplemental Table 2).

## Results

### Characteristics of the study population

Overall, the study population comprised more women than men (58% vs 42%) and the median age was approximately 66 years (IQR: 59–74), most of the participants had a lower/intermediate education (68.9%) and median BMI was 26.5 kg/m^2^ (IQR: 24.3–29.1). Participants attending in autumn were about 3 years older than those who attended in summer, and a larger consumption of energy intake was observed in autumn than in summer. Participants with comorbidities were more likely to attend in winter than in summer (Table 2).ù

**Table 2:** Characteristics of the study population (*n* = 12,589 observations) stratified by season

Characteristics	Overall	*N*	Winter	*N*	Spring	*N*	Summer	*N*	Autumn	*N*	*P* value*
	Median (IQR)		Median (IQR)		Median (IQR)		Median (IQR)		Median (IQR)		
Age (years)	66.3 (59.4–73.7)	12,589	66.3 (59.8–73.5)	3239	66.5 (58.8 to 74.1)	3951	67.3 (59.7–74.5)	2868	64.7 (59.3–72.5)	2531	< **0.01**
Physical activity (z score MET-hours/week)^a^	− 0.22 (− 0.77 to 0.58)	10,412	− 0.19 (− 0.75 to 0.55)	2643	− 0.26 (− 0.78 to 0.48)	3301	− 0.23 (− 0.78 to 0.56)	2388	− 0.11 (− 0.77 to 0.78)	2080	< **0.01**
BMI	26.5 (24.3–29.1)	12,397	26.3 (24.2–29.0)	3204	26.6 (24.3–29.3)	3882	26.4 (24.2–29.0)	2820	26.4 (24.3–29.1)	2491	**0.04**
Energy intake (kilocalories/day)	1996 (1653–2392)	12,589	2001 (1671–2396)	3239	1984 (1654–2391)	3951	1982 (1625–2371)	2868	2021 (1657–2416)	2531	0.06
Diet quality score	7 (5–8)	12,589	7 (6–8)	3239	7 (5–8)	3951	7 (5–8)	2868	7 (6–8)	2531	0.63

	*N*	%	*N*	%	*N*	%	*N*	%	*N*	%	
Sex											
Men	5306	42.1	1391	43.0	1679	42.5	1181	41.18	1055	41.7	0.50
Women	7283	57.9	1848	57.0	2272	57.5	1687	58.82	1476	58.3	
Education											
Primary	1695	13.6	410	12.8	550	14.0	409	14.35	326	13.0	< **0.01**
Lower	5189	41.6	1370	42.6	1553	39.7	1196	41.96	1070	42.8	
Intermediate	3626	29.0	911	28.4	1128	28.8	828	29.05	759	30.4	
Higher	1968	15.8	521	16.2	685	17.5	417	14.63	345	13.8	
Smoking status											
Never	4031	32.1	1046	32.5	1261	32.0	908	31.76	816	32.5	0.42
Ever	5879	46.9	1537	47.8	1855	47.0	1345	47.04	1142	45.4	
Current	2626	21.0	635	19.7	829	21.0	606	21.20	556	22.1	
Prevalent diseases^b^											
Yes	2155	17.1	590	18.2	692	17.5	476	16.60	397	15.7	0.06
No	10434	82.9	2649	81.8	3259	82.5	2392	83.40	2134	84.3	

Bold values are considered statistically significant (*P* < 0.05)

Some characteristics do not sum up, because table is based on non-imputed data

*BMI* body mass index, *MET* metabolic equivalent of task

*P value of population differences between seasons estimated using Kruskal–Wallis test for continuous variables, and Chi-square test for categorical variables

^a^Physical activity is based on non-imputed data, because (imputed) standardized values of physical activity were used for the analyses

^b^Prevalent diseases include stroke, myocardial infarction (MI), diabetes mellitus type 2 (T2DM), and cancer

### Seasonality of diet quality and daily intake of food groups

**Fig. 1:**
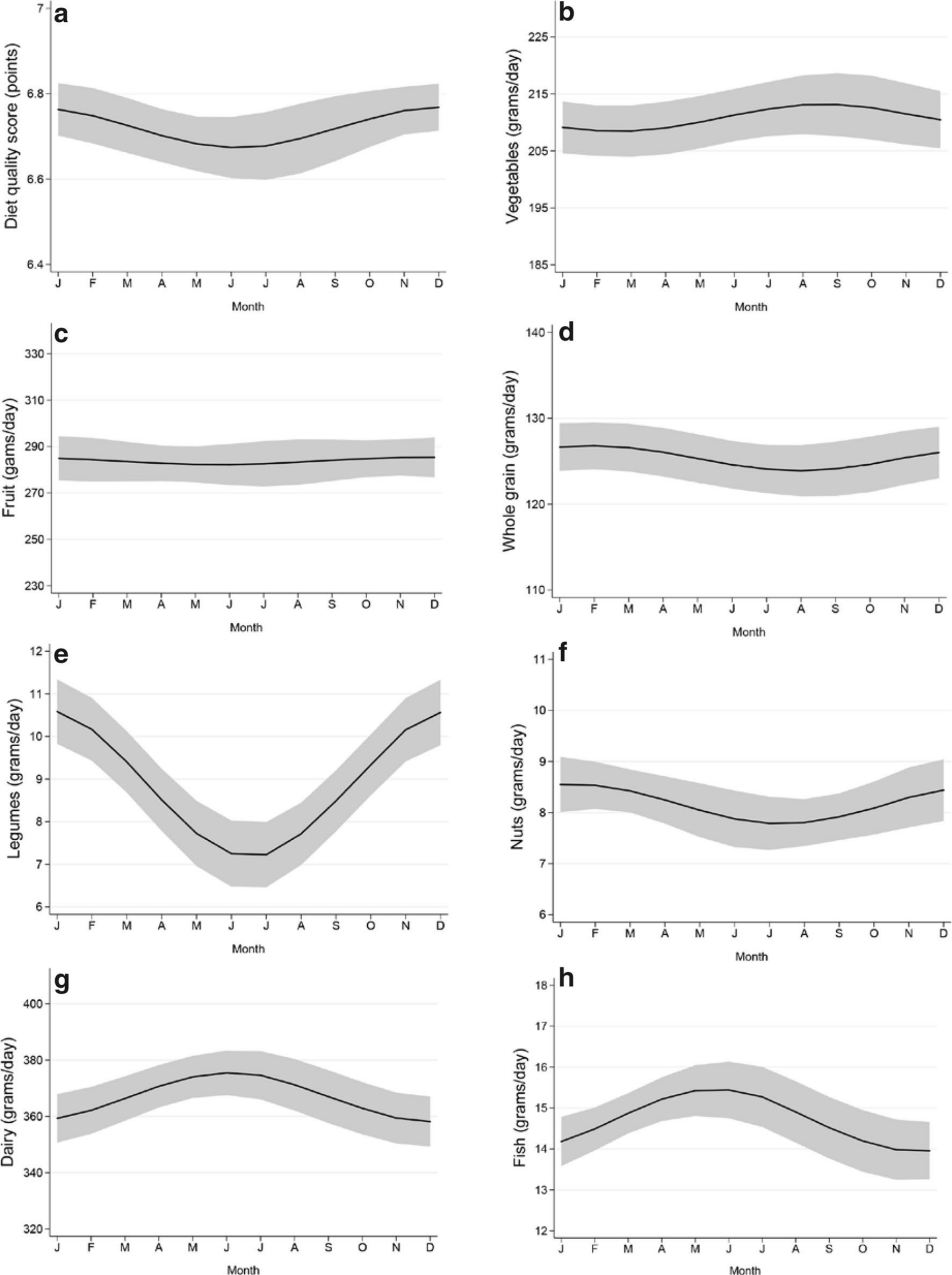

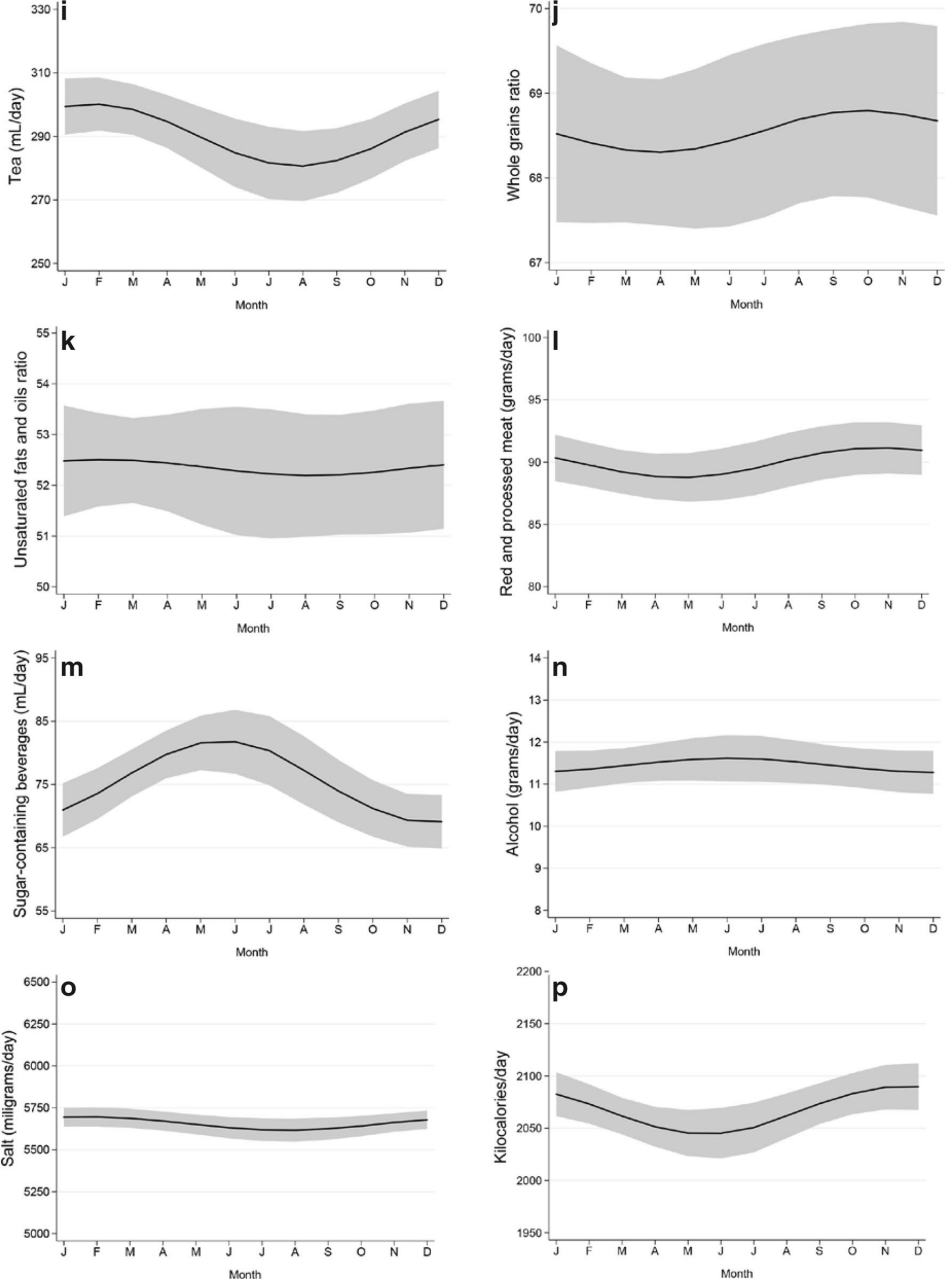
**a**–**p** Seasonal variation of diet quality and food groups. Graphical representation of the seasonal variation of the diet quality score and food groups intake. The gray area represents the 95% confidence interval around the pattern. Estimates are adjusted for cosinor terms, age, sex, cohort, (kilocalories), physical activity, smoking behavior, BMI, diseases and education

Diet quality had a significant seasonality with a peak in December (seasonal variation = 0.10, 95% CI: 0.01 to 0.18), indicating a higher adherence to guidelines in winter than in summer. Seasonal variation was observed for intake of legumes, nuts, tea, red and processed meat, salt and amount of energy, with a winter peak; and for sugar-containing beverages, dairy, and fish intake, with a summer peak (Fig. 1 Seasonal variation of diet quality and food groups). The largest seasonal variation was observed for legumes, with an intake of up to 3.5 g/day higher in winter than in summer, which represented 39% of the average legume intake (9.1 g/day) in our population (Table 3). No large seasonality was observed for intake of vegetables, fruits, whole grain products, whole grain ratio, unsaturated fat and oil ratio, or alcohol. Results were similar when using the non-imputed dataset (Supplemental Table 2).

**Table 3:** Seasonality of the diet quality score and of each contributing food group, *n* = 12,589 observations

Outcome	Model	Seasonal variation*	95% Confidence interval	Mean daily score/intake	Seasonal variation in percentages^a^	Peak	Nadir
Diet quality score (0–14)	Model 1	0.12	**0.03–0.21**				
	Model 2	0.10	**0.01–0.18**	6.72	1.49	19-Dec	19-Jun
Kilocalories/day	Model 1	45.93	**17.92–73.95**				
	Model 2	46.03	**18.27–73.80**	2067.43	2.23	29-Nov	30-May
Food groups							
Vegetables (g/day)	Model 1	4.67	− 2.19–11.54				
	Model 2	4.81	− 1.96–11.58	210.09	2.29	1-Sep	2-Mar
Fruits (g/day)	Model 1	6.57	− 5.360–18.50				
	Model 2	3.18	− 8.63–14.99	284.84	1.12	3-Dec	4-Jun
Whole grain (g/day)	Model 1	2.95	− 0.37–6.27				
	Model 2	2.95	− 0.37–6.27	125.36	2.35	12-Feb	12-Aug
Legumes (g/day)	Model 1	3.52	**2.62–4.42**				
	Model 2	3.51	**2.61–4.41**	9.09	38.61	30-Dec	30-Jun
Nuts (g/day)	Model 1	0.82	**0.20–1.45**				
	Model 2	0.78	**0.16–1.41**	8.25	9.45	25-Jan	26-Jul
Dairy (g/day)	Model 1	16.95	**5.03–28.87**				
	Model 2	17.52	**5.60–29.44**	365.65	4.79	17-Jun	16-Dec
Fish (g/day)	Model 1	1.45	**0.57–2.33**				
	Model 2	1.52	**0.64–2.40**	14.82	10.26	1-Jun	30-Nov
Tea (mL/day)	Model 1	21.48	**9.21–33.76**				
	Model 2	19.82	**7.65–32.00**	288.42	6.87	9-Feb	9-Aug
Whole grain ratio	Model 1	0.59	− 0.66–1.83				
	Model 2	0.50	− 0.75–1.75	68.54	0.73	10-Oct	10-Apr
Unsaturated fat and oil ratio	Model 1	0.45	− 1.00–1.91				
	Model 2	0.32	− 1.16–1.79	52.41	0.61	19-Feb	19-Aug
Red and processed meat (g/day)	Model 1	2.11	− 0.26–4.47				
	Model 2	2.43	**0.11–4.75**	89.70	2.71	4-Nov	6-May
Sugar-containing beverages (mL/day)	Model 1	13.01	**7.22–18.80**				
	Model 2	12.96	**7.16–18.77**	75.52	17.16	1-Jun	1-Dec
Alcohol (g/day)	Model 1	0.41	− 0.25–1.07				
	Model 2	0.34	− 0.31–0.99	11.47	2.96	16-Jun	16-Dec
Salt (mg/day)	Model 1	84.87	**18.37–151.38**				
	Model 2	80.70	**14.50–146.90**	5658.87	1.43	5-Feb	5-Aug

Bold coefficients are statistically significant at 95% confidence level

Model 1 includes cosinor terms, sex, age, cohort and energy intake

Model 2 additionally adjusted for physical activity, smoking behavior, body mass index, prevalent diseases (stroke, myocardial infarction, diabetes mellitus type 2, and cancer), and education

*Seasonal variation = maximum difference between the highest annual average (peak) and lowest annual average (nadir)

^a^Seasonal variation in percentages (seasonal variation/mean daily score or intake × 100%)

The one by one exclusion of food groups showed that the seasonality of overall diet quality was mainly driven by the seasonality of legumes. Diet quality seasonality was reduced by 80% after excluding legumes from the score, by 40% after excluding fruit, and by 30% after excluding nuts. In contrast, diet quality seasonality increased by 30% and 20% after excluding dairy and vegetables from the score, respectively (Table 4).

**Table 4:** Seasonal variation of diet quality score excluding one food group at a time, n = 12,589 observations

Outcome	Seasonal variation*	95% Confidence interval	%^a^
Diet quality score	0.10	**0.01–0.18**	
Diet quality score excluding			
Vegetables	0.12	**0.04–0.20**	+ 20
Fruit	0.06	− 0.02–0.13	− 40
Whole grain products	0.10	**0.02–0.18**	0
Legumes	0.02	− 0.06–0.10	− 80
Nuts	0.07	− 0.01–0.16	− 30
Dairy	0.13	**0.05–0.21**	+ 30
Fish	0.11	**0.03–0.19**	+ 10
Tea	0.08	− 0.00–0.17	− 20
Whole grain ratio	0.09	**0.01–0.17**	− 10
Unsaturated fat and oil ratio	0.10	**0.02–0.18**	0
Red and processed meat	0.11	**0.03–0.20**	+ 10
Sugar-containing beverages	0.07	− 0.01–0.15	− 30
Alcohol	0.09	**0.01–0.17**	− 10
Salt	0.11	**0.03–1.19**	+ 10

Bold coefficients are statistically significant at 95% confidence level

Estimates are adjusted for cosinor terms, sex, age, cohort, energy intake, physical activity, smoking behavior, body mass index, prevalent diseases (stroke, myocardial infarction, diabetes mellitus type 2, and cancer), and education

*Seasonal variation = maximum difference between the highest annual average (peak) and lowest annual average (nadir)

^a^Percentage reduction or increment of the seasonal variation by excluding food groups, compared to the total diet score (SV − 0.10/(0.10 × 100%))

### Subgroup analyses

Diet quality and more food groups had a larger seasonal variation among men, participants with BMI > 25 kg/m^2^, those living with relatives/others, and participants with high SES, than among their respective counterparts. No large differences in seasonal pattern were observed according to age group or FFQ used. (Supplemental Tables 3, 4).

Participants with a lower overall diet quality were more likely to be men, lower educated, current or ever smokers, were more often having comorbidities, and living with relatives. In addition, participants with a lower diet quality had a lower energy intake (Supplemental Table 5).

## Discussion

In this Dutch population, diet quality had a seasonal pattern with a peak, i.e., better diet quality, in winter. This pattern was mostly explained by the peak of legumes, tea, and nuts, and the nadir of SCBs intake in winter. Dairy and fish consumption showed a peak in summer–autumn season, which explains a shift towards a reduced magnitude of the seasonality of total diet quality. A larger seasonality in more food groups and a lower diet quality was observed among men, subjects with a higher BMI, higher SES, and those living with a partner or relatives, than among their respective counterparts.

Diet quality increased in winter, mostly due to the winter peak of legumes and nuts intake and to the summer peak (and winter nadir) of dairy and sugar-containing beverage intake. The winter peak of legumes intake has been previously reported [11, 45], and is likely explained by the preference among Dutch population to consume legume-based dishes during the winter, such as lentil- and split pea soup. We are not aware of comparable studies addressing the seasonality of nuts intake, although people could prefer them in colder months for its fat content. The summer peak of sugar-containing beverages intake has also been reported before [46, 47], and is attributed to the preference for sweet refreshing beverages in summer. Probably, these are replaced in winter by warmer beverages, such as tea and coffee, as we and others [47] found. Finally, the summer peak of dairy intake is consistent with one study performed among Spanish men, but not among Finnish women [11, 47]. In our population, the pattern could be attributed to the increment of ice creams intake in summer.

Interestingly, the seasonal pattern was also modified by vegetable and fruit intake, which did not show a significant seasonality. We hypothesize that the exclusion of vegetables from the score reveals the pattern of a lower diet quality, which is less stable throughout the year. Indeed, diet quality and vegetable intake among people who regularly eat vegetables may be less influenced by season because of diet consciousness. As for fruit intake, we hypothesize that those who do not eat fruits regularly are more likely to eat it along with other food groups with a strong seasonal pattern, e.g., legumes and nuts. The stable intake of vegetables and fruits throughout the year in our study opposes the seasonality observed in previous studies [10, 11, 45, 46], and could be attributed to the constant availability of affordable vegetables and fruits in the Netherlands [48]. However, because only 50% of our population met the guidelines for vegetable and fruit intake [22], aiming to increase the intake of vegetables and fruits may contribute to enhance overall diet quality.

Overall, a larger seasonality was observed in those food groups for which less people followed the intake guideline recommendations (i.e., fish, tea, nuts and legumes). For these food groups, intake was below the recommendations in more than 60% of the participants [22]. This suggests that addressing the mechanisms underlying the large seasonal variation of these food groups could contribute to improve the adherence to guideline recommendations.

Seasonality of alcohol intake appears also influenced by age. In contrast with previous studies showing a summer peak of alcohol intake among younger population [11], we did not find such variation in our study. Arguably, our middle-aged and elderly population would be less inclined to increase their alcohol intake during summer activities.

A larger seasonality in diet quality and in more food groups was observed among men and among participants with high BMI than in their counterparts. The sex differences in the seasonality of food groups are in agreement with previous studies [9, 11, 46], and can be explained by a better diet consciousness among women [22, 49]. A better diet consciousness could also explain the more stable diet quality of participants with lower BMI. Interestingly, participants with higher SES and subjects living with a partner or relatives exhibited a larger seasonality of food groups’ intake than their corresponding counterparts. However, this pattern appears to reflect that of men, as the proportion of men was higher among participants with higher SES and those living with a partner or relatives. The larger seasonality of food groups’ consumption among participants with high SES also contradicted our working hypothesis about the role of the price of food products on the seasonality of diet [50], which would lead to a larger seasonality in the lower SES group. However, it is possible that those in the lower SES group replace food items with other less expensive within the same food group, or that they purchase food items without prices varying seasonally. These hypotheses need to be tested in other populations with different distribution of SES.

Taken together, our findings suggest that policies aimed to improve diet quality could address the seasonal factors leading to a lower intake of legumes, nuts, and tea in summer and of fish in winter. Although the availability of certain food groups might vary according to their natural season, the seasonality of the food groups appears to have more cultural and behavioral mechanisms underlying. Stakeholders can collaborate with markets and food producers to make certain food groups more attractive when the intake is anticipated to decline. For example, legumes intake could be promoted during summer with legume-based salads or other palatable recipes containing legumes. Also, fish intake could be promoted to replace red and processed meat, which appeared strongly ingrained in our population diet. Indeed, less than 20% of the participants reported an intake of red and processed meat below 300 g/w, and the intake had a small seasonality. In contrast, fish intake had a summer-peak that coincided not only with the period of lowest intake of red and processed meat, but also with the traditional Dutch herring season. Therefore, the factors underlying the summer preference for fish could be accounted for to increase the intake in other seasons. Finally, the summer-peak of sugar-containing beverages intake can be reduced by aiming to replace it by other non-sugar-containing beverages during summer activities.

Several strengths of this study are worth mentioning. To our knowledge, we are the first to address the seasonality of diet quality and to examine the food groups that influence this pattern. In addition, we used validated FFQs to determine dietary intake [16, 17]. Furthermore, our study uses data from a large population-based study and is representative of the general adult and elderly population, and we accounted for the non-randomness of the participation over the season by adjusting for several covariates. However, some limitations need to be acknowledged. First, we used two different FFQs to assess diet quality; one asks about dietary intake in the past year and the other requests for the intake of the last month. However, this had a small impact in our findings, as these remained similar in the stratified analysis according to FFQ. Nevertheless, the fact that the seasonality estimates remained similar in the stratified analysis suggests that people are more likely to report their current diet behavior than the actual average during the last year [51]. Therefore, researchers addressing the long-term diet behavior need to account for this limitation, especially in geographic areas with seasonal variation. Second, the use of the FFQ to measure dietary intake, instead of 24-h dietary recalls or dietary records to avoid recalling bias could have led to an underestimation of the actual seasonality. Third, we were able to include up to two repeated measurements per participant, which reduced the within-subject variation of our seasonality estimates. It would be valuable to conduct a similar study using dietary record methods with more measurements per person during different seasons to improve the understanding of the seasonal patterns.

In conclusion, diet quality has a significant seasonality, with specific food groups counteractively contributing to this pattern. The pattern was mostly explained by the seasonality in intake of legumes, sugar-containing beverages, tea, dairy and nuts. Men and those with highest BMI had the largest seasonality of diet quality and food groups’ intake throughout the year. Season should be accounted for when measuring diet quality. Reducing the seasonality in the intake of the food groups with largest seasonality could contribute to improve the adherence to intake guideline recommendations, and arguably, to improve the overall diet quality.

## Electronic supplementary material

Below is the link to the electronic supplementary material.

Supplementary material 1 (PDF 482 KB)

## Data Availability

Data can be obtained upon request. Requests should be directed towards the management team of the Rotterdam Study (secretariat.epi@erasmusmc.nl), which has a protocol for approving data requests. Data cannot be made freely available on a public source considering privacy restrictions and regulations.
