# Supplementary material for: Seasonal variation of diet quality in a large middle-aged and elderly Dutch population-based cohort
Source: Eur J Nutr. 2019 Feb 8;59(2):493–504. doi: 10.1007/s00394-019-01918-5 (PMC7058580; doi:10.1007/s00394-019-01918-5)
Supplement: Supplementary file 1 — Supplementary material 1 (PDF 482 KB) [file 394_2019_1918_MOESM1_ESM.pdf]

Online Supplementary Material

Seasonal variation of diet quality in a large middle-aged and elderly Dutch population-based cohort
European Journal of Nutrition

Janine E. van der Toorn*; Magda Cepeda*; Jessica C. Kiefte de Jong; Oscar H. Franco; Trudy Voortman; Josje D. Schoufour

*Both authors contributed equally

[1] Department of Epidemiology, Erasmus MC, University Medical Centre, Rotterdam, the Netherlands

[2] Leiden University College, The Hague, the Netherlands.

Corresponding author: Magda Cepeda, Department of Epidemiology, Erasmus MC

Office Na-2911, PO Box 2040, 3000 CA Rotterdam, the Netherlands

Phone: +31 10 70 38910; Fax: +31 10 70 44657; Email: m.cepedagil@erasmusmc.nl

Contents

Supplemental Figure 1. Appendix Flowchart of sample size selection 2

Supplemental Table 1. STROBE Statement—Checklist of items that should be included in reports of cross-sectional studies 3

Supplemental Table 2. Seasonal variation of diet quality score and of contributing food group, based on non-imputed dataset. 5

Supplemental Table 3a. Stratified analyses: Seasonal variation of diet quality score and of each contributing food group according to sex 6

Supplemental Table 3b. Stratified analyses: Seasonal variation of diet quality score and of each contributing food group according to age group 7

Supplemental Table 3c. Stratified analyses: Seasonal variation of diet quality score and of each contributing food group according to age group 8

Supplemental Table 3d. Stratified analyses: Seasonal variation of diet quality score and of each contributing food group according to socioeconomic status 9

Supplemental Table 3e. Stratified analyses: Seasonal variation of diet quality score and of each contributing food group according to living status 10

Supplemental Table 4. Stratified analyses: Seasonal variation of diet quality according to FFQ 11

Supplemental Table 5. Characteristics of the study population stratified by participants with a lower, intermediate, and higher diet quality score 12

Online Supplementary Material

Supplemental Figure 1. Appendix Flowchart of sample size selection

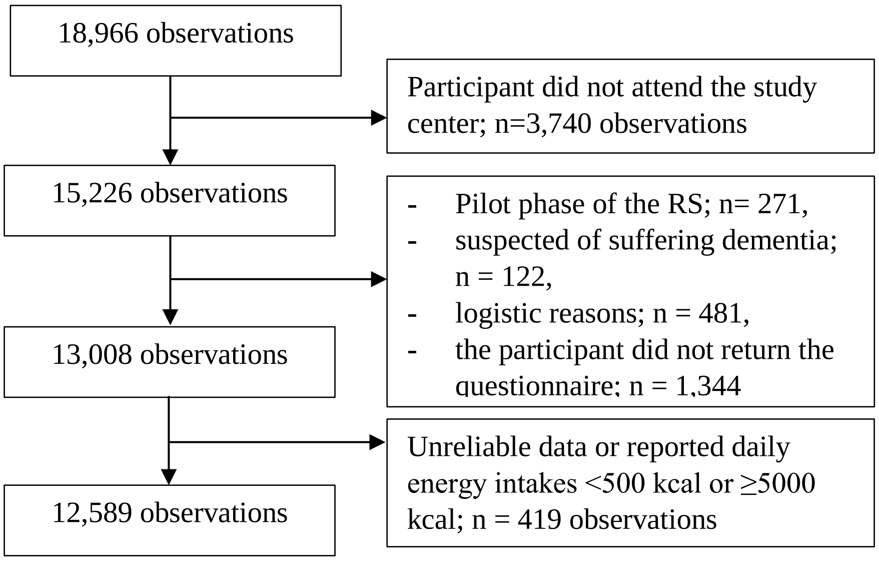

Imputation procedures: Sequential multiple imputation using chained equations was performed to impute missing values only of covariates. The predictors used to impute the covariates were age, sex, cohort, standardized MET hours/week, smoking behavior, BMI, education, diet quality score. We performed the imputation stratified per number of visits, in order to use the data from other visits for those participants with more than one visit. For ordered categorical variables (education) we used an ordered logit function. For categorical non-ordered variables (smoking behavior) we used multinomial logit function. For BMI and physical activity, we used linear functions. To ensure reproducibility, we used a random seed (2005). We created five imputed datasets. Covariates with missing values were: education: 111missing, smoking behavior: 53missing, BMI: 192missing, physical activity: 2177missing. Imputations were performed using the *mi impute* command of Stata software.

Online Supplementary Material

Supplemental Table 1. STROBE Statement—Checklist of items that should be included in reports of cross-sectional studies

	Item No	Recommendation	Page
**Title and abstract**	1	(*a*) Indicate the study's design with a commonly used term in the title or the abstract	1
		(*b*) Provide in the abstract an informative and balanced summary of what was done and what was found	1
**Introduction**			
Background/rationale	2	Explain the scientific background and rationale for the investigation being reported	3
Objectives	3	State specific objectives, including any prespecified hypotheses	3
**Methods**			
Study design	4	Present key elements of study design early in the paper	4
Setting	5	Describe the setting, locations, and relevant dates, including periods of recruitment, exposure, follow-up, and data collection	4
Participants	6	(*a*) Give the eligibility criteria, and the sources and methods of selection of participants	4
Variables	7	Clearly define all outcomes, exposures, predictors, potential confounders, and effect modifiers. Give diagnostic criteria, if applicable	4-6
Data sources/ measurement	8*	For each variable of interest, give sources of data and details of methods of assessment (measurement). Describe comparability of assessment methods if there is more than one group	4-6
Bias	9	Describe any efforts to address potential sources of bias	6-7
Study size	10	Explain how the study size was arrived at	4
Quantitative variables	11	Explain how quantitative variables were handled in the analyses. If applicable, describe which groupings were chosen and why	4-5
Statistical methods	12	(*a*) Describe all statistical methods, including those used to control for confounding	5-7
		(*b*) Describe any methods used to examine subgroups and interactions	5-7
		(*c*) Explain how missing data were addressed	6
		(*d*) If applicable, describe analytical methods taking account of sampling strategy	NA
		(*e*) Describe any sensitivity analyses	6
**Results**			
Participants	13*	(a) Report numbers of individuals at each stage of study—eg numbers potentially eligible, examined for eligibility, confirmed eligible, included in the study, completing follow-up, and analysed	4, Supplemental Figure 1
		(b) Give reasons for non-participation at each stage	Supplemental Figure 1
		(c) Consider use of a flow diagram	Supplemental Figure 1
Descriptive data	14*	(a) Give characteristics of study participants (eg demographic, clinical, social) and information on exposures and potential confounders	8, Table 2
		(b) Indicate number of participants with missing data for each variable of interest	Supplemental Figure 1
Outcome data	15*	Report numbers of outcome events or summary measures	Table 2
Main results	16	(*a*) Give unadjusted estimates and, if applicable, confounder-adjusted estimates and their precision (eg, 95% confidence interval). Make clear which confounders were adjusted for and why they were included	Table 3
		(*b*) Report category boundaries when continuous variables were categorized	NA
		(*c*) If relevant, consider translating estimates of relative risk into absolute risk for a meaningful time period	NA
Other analyses	17	Report other analyses done—eg analyses of subgroups and interactions, and	Table 4, Supplemental

		sensitivity analyses	Table 3a-e, 4 and 5
**Discussion**			
Key results	18	Summarise key results with reference to study objectives	9
Limitations	19	Discuss limitations of the study, taking into account sources of potential bias or imprecision. Discuss both direction and magnitude of any potential bias	11
Interpretation	20	Give a cautious overall interpretation of results considering objectives, limitations, multiplicity of analyses, results from similar studies, and other relevant evidence	9-11
Generalisability	21	Discuss the generalisability (external validity) of the study results	11
**Other information**			
Funding	22	Give the source of funding and the role of the funders for the present study and, if applicable, for the original study on which the present article is based	13

*Give information separately for exposed and unexposed groups.

Note: An Explanation and Elaboration article discusses each checklist item and gives methodological background and published examples of transparent reporting. The STROBE checklist is best used in conjunction with this article (freely available on the Web sites of PLoS Medicine at http://www.plosmedicine.org/, Annals of Internal Medicine at http://www.annals.org/, and Epidemiology at http://www.epidem.com/). Information on the STROBE Initiative is available at www.strobe-statement.org.

Online Supplementary Material

Supplemental Table 2. Seasonal variation of diet quality score and of contributing food group, based on non-imputed dataset.

Outcome	Model	Seasonal variation ***	95%-confidence interval	Peak	Nadir	Observations
Diet quality score (0-14)	Model 1	0.12	**0.03 – 0.21***	14-Dec	14-Jun	12,589
	Model 2	0.15	**0.06 – 0.25***	14-Dec	14-Jun	10,173
Kilocalories/day	Model 1	45.93	**17.92 – 73.95***	20-Nov	22-May	12,589
	Model 2	43.58	**13.08 – 74.07***	12-Dec	12-Jun	10,173
Food groups						
Vegetables (g/d)	Model 1	4.67	-2.19 – 11.54	13-Sep	14-Mar	12,589
	Model 2	6.46	-1.23 – 14.15	30-Sep	31-Mar	10,173
Fruits (g/d)	Model 1	6.57	-5.36 – 18.50	2-Dec	3-Jun	12,589
	Model 2	1.54	-12.08 – 15.16	27-Nov	28-May	10,173
Wholegrain (g/d)	Model 1	2.95	-0.37 – 6.27	7-Feb	8-Aug	12,589
	Model 2	3.51	-0.17 – 7.19	2-Mar	31-Aug	10,173
Legumes (g/d)	Model 1	3.52	**2.62 – 4.42***	29-Dec	29-Jun	12,589
	Model 2	4.01	**2.95 – 5.07***	26-Dec	26-Jun	10,173
Nuts (g/d)	Model 1	0.82	**0.20 – 1.45***	25-Jan	25-Jul	12,589
	Model 2	1.11	**0.40 – 1.81***	11-Jan	12-Jul	10,173
Dairy (g/d)	Model 1	16.95	**5.03 – 28.87***	15-Jun	15-Dec	12,589
	Model 2	16.67	**3.58 – 29.75***	12-Jun	11-Dec	10,173
Fish (g/d)	Model 1	1.45	**0.57 – 2.33***	2-Jun	2-Dec	12,589
	Model 2	1.23	**0.25 – 2.22***	10-Jun	9-Dec	10,173
Tea (mL/d)	Model 1	21.48	**9.21 – 33.76***	6-Feb	6-Aug	12,589
	Model 2	19.65	**6.05 – 33.25***	7-Feb	7-Aug	10,173
Whole grains ratio	Model 1	0.59	-0.66 – 1.83	13-Oct	13-Apr	12,589
	Model 2	0.40	-0.90 – 1.69	25-Jan	25-Jul	10,173
Unsaturated fats and oils ratio	Model 1	0.45	-1.00 – 1.91	19-Jan	20-Jul	12,589
	Model 2	0.81	-0.82 – 2.45	28-Nov	29-May	10,173
Red and processed meat (g/d)	Model 1	2.11	-0.26 – 4.47	31-Oct	1-May	12,589
	Model 2	1.93	-0.65 – 4.50	28-Oct	29-Apr	10,173
Sugar-containing beverages (mL/d)	Model 1	13.01	**7.22 – 18.80**	2-Jun	2-Dec	12,589
	Model 2	12.14	**5.56 – 18.73**	1-Jun	1-Dec	10,173
Alcohol (g/d)	Model 1	0.41	-0.25 – 1.07	4-Jun	4-Dec	12,589
	Model 2	0.71	-0.02 – 1.44	1-Jun	1-Dec	10,173
Salt (mg/d)	Model 1	84.87	**18.37 – 151.28***	4-Feb	5-Aug	12,589
	Model 2	93.17	**19.20 – 167.15***	15-Jan	15-Jul	10,173

Model1 includes cosinor terms, sex, age, cohort and energy intake

Model 2 additionally adjusted for physical activity, smoking behaviour, body mass index, prevalent diseases (stroke, myocardial infarction, diabetes mellitus type 2, and cancer), and education.

** Seasonal variation = maximum difference between the highest annual average (peak) and lowest annual average (nadir)

* Statistically significant

Online Supplementary Material

Supplemental Table 3a. Stratified analyses: Seasonal variation of diet quality score and of each contributing food group according to sex

	Men n=5,306 observations					Women n=7,283 observations				
**Outcome**	**Seasonal variation****	**%[1]**	**95%-confidence interval**	**Peak**	**Nadir**	**Seasonal variation****	**%[1]**	**95%-confidence interval**	**Peak**	**Nadir**
**Diet quality score (0-14)**	0.11	1.73	-0.02 – 0.24	6-Jan	6-Jul	0.09	1.29	-0.02 – 0.21	4-Dec	4-Jun
**Kilocalories/day**	53.84	2.34	**7.34 – 100.33***	13-Nov	15-May	41.82	2.20	**8.17 – 75.47***	14-Dec	14-Jun
Food groups										
**Vegetables (g/d)**	11.39	5.55	**1.68 – 21.09***	17-Sep	18-Mar	2.33	1.09	-6.90 – 11.57	16-Jun	16-Dec
**Fruit (g/d)**	6.95	2.76	-11.30 – 25.21	1-May	31-Oct	10.49	3.40	-5.46 – 26.45	11-Nov	12-May
**Whole grain products (g/d)**	4.54	3.22	-1.25 – 10.32	27-Dec	27-Jun	3.73	3.28	-0.23 – 7.68	24-Mar	23-Sep
**Legumes (g/d)**	4.12	39.47	**2.59 – 5.65***	23-Dec	24-Jun	3.13	38.60	**2.05 – 4.22***	4-Jan	5-Jul
**Nuts (g/d)**	0.62	6.11	-0.44 – 1.68	29-Jan	30-Jul	0.96	13.99	**0.21 – 1.72***	27-Jan	28-Jul
**Dairy (g/d)**	28.96	8.00	**9.72 – 48.20***	11-Jun	11-Dec	10.46	2.84	-4.59 – 25.50	27-Jun	27-Dec
**Fish (g/d)**	2.47	15.50	**1.02 – 3.92***	22-May	20-Nov	0.90	6.43	-0.18 – 1.98	22-Jun	22-Dec
**Tea (mL/d)**	26.54	10.70	**8.99 – 44.09***	18-Feb	19-Aug	15.98	5.03	-0.64 – 32.61	28-Jan	28-Jul
**Unsaturated fats and oils ratio**	0.18	0.26	-1.76 – 2.13	5-May	4-Nov	1.01	1.47	-0.59 – 2.61	6-Oct	6-Apr
**Whole grains ratio**	0.47	0.89	-1.73 – 2.67	17-Feb	17-Aug	0.25	0.48	-1.74 – 2.24	26-Feb	27-Aug
**Red and processed meat (g/d)**	0.82	0.80	-3.05 – 4.69	21-Nov	22-May	3.55	4.43	**0.73 – 6.38***	28-Oct	28-Apr
**Sugar-containing beverages (mL/d)**	17.66	20.31	**7.91 – 27.41***	14-Jun	14-Dec	9.97	14.84	**2.89 – 17.06***	16-May	15-Nov
**Alcohol (g/d)**	0.59	3.51	-0.69 – 1.87	28-May	27-Nov	0.30	3.96	-0.34 – 0.94	28-Jul	27-Jan
**Salt (mg/d)**	112.21	1.79	-0.78 – 225.19	19-Jan	20-Jul	68.83	1.32	-11.25 – 148.92	28-Feb	29-Aug

Seasonal variation adjusted for cosinor terms, age, sex, cohort, physical activity, smoking behaviour, body mass index, comorbidities, education, and kilocalories/day (except when used as outcome).

** Seasonal variation expresses the maximum difference between the average highest intake/score (peak) and average lowest intake/score (nadir)

* Seasonal variation is statistically significant

[1] (Seasonal variation/average intake or score)*100%

Online Supplementary Material

Supplemental Table 3b. Stratified analyses: Seasonal variation of diet quality score and of each contributing food group according to age group

	<65 years old n=5,544 observations					≥65 years old n=7,045 observations				
**Outcome**	**Seasonal variation****	**%[1]**	**95%-confidence interval**	**Peak**	**Nadir**	**Seasonal variation****	**%[1]**	**95%-confidence interval**	**Peak**	**Nadir**
Diet quality score (0-14)	0.13	1.94	-0.01 – 0.26	9-Dec	10-Jun	0.10	1.48	-0.01 – 0.22	2-Feb	3-Aug
Kilocalories/day	76.06	3.49	**31.70 – 120.43***	18-Nov	19-May	33.01	1.67	-3.54 – 69.57	6-Dec	7-Jun
Food groups										
Vegetables (g/d)	7.93	3.42	-3.10 – 18.96	12-Sep	13-Mar	2.38	1.23	-6.47 – 11.23	7-Jun	6-Dec
Fruit (g/d)	8.57	3.18	-7.70 – 24.84	21-Jan	22-Jul	5.24	1.76	-11.09 – 21.56	5-Mar	4-Sep
Whole grain products (g/d)	5.19	3.80	-0.16 – 10.54	9-Dec	9-Jun	3.88	3.33	-0.50 – 8.26	18-Mar	17-Sep
Legumes (g/d)	3.63	35.62	**2.24 – 5.02***	26-Nov	27-May	4.15	50.46	**2.94 – 5.36***	23-Jan	23-Jul
Nuts (g/d)	0.65	6.59	-0.38 – 1.67	1-Feb	2-Aug	0.94	13.48	**0.14 – 1.75***	10-Feb	10-Aug
Dairy (g/d)	28.26	7.59	**8.74 – 47.79***	10-Jun	10-Dec	13.70	3.80	-1.66 – 29.06	31-Jul	30-Jan
Fish (g/d)	0.96	6.27	-0.37 – 2.28	25-May	24-Nov	1.94	13.45	**0.70 – 3.18***	31-May	30-Nov
Tea (mL/d)	22.07	8.48	**2.66 – 41.47***	9-Feb	10-Aug	12.78	4.12	-3.74 – 29.31	3-Jan	4-Jul
Unsaturated fats and oils ratio	1.65	2.41	-0.16 – 3.45	2-Dec	2-Jun	0.60	0.87	-1.08 – 2.27	31-Jul	31-Jan
Whole grains ratio	1.12	2.06	-0.97 – 3.21	21-Jan	22-Jul	2.05	4.04	0.00 – 4.10	31-Mar	30-Sep
Red and processed meat (g/d)	3.74	3.94	**0.10 – 7.38***	21-Sep	23-Mar	3.52	4.11	**0.44 – 6.60***	17-Nov	18-May
Sugar-containing beverages (mL/d)	13.39	15.23	**4.16 – 22.63***	1-Jul	0-Jan	12.27	18.66	**4.99 – 19.56***	24-May	23-Nov
Alcohol (g/d)	0.40	3.01	-0.84 – 1.65	17-Apr	17-Oct	0.40	3.99	-0.44 – 1.24	6-Jul	5-Jan
Salt (mg/d)	104.50	1.72	-1.72 – 210.73	24-Nov	26-May	99.97	1.88	**12.87 – 187.06***	7-Mar	6-Sep

Seasonal variation adjusted for cosinor terms, age, sex, cohort, physical activity, smoking behaviour, body mass index, comorbidities, education, and kilocalories/day (except when used as outcome).

** Seasonal variation expresses the maximum difference between the average highest intake/score (peak) and average lowest intake/score (nadir)

* Seasonal variation is statistically significant

[1] (Seasonal variation/average intake or score)*100%

Online Supplementary Material

Supplemental Table 3c. Stratified analyses: Seasonal variation of diet quality score and of each contributing food group according to age group

	BMI 18.5 – 25 kg/m^2 n=2,714 observations					BMI >25 kg/m^2 n=9,799 observations				
**Outcome**	**Seasonal variation****	**%[1]**	**95%-confidence interval**	**Peak**	**Nadir**	**Seasonal variation****	**%[1]**	**95%-confidence interval**	**Peak**	**Nadir**
Diet quality score (0-14)	0.02	0.29	-0.17 – 0.21	4-Jun	3-Dec	0.13	1.95	**0.04 – 0.23***	22-Dec	22-Jun
Kilocalories/day	72.57	3.45	**15.67 – 129.46***	7-Jan	7-Jul	44.87	2.18	**12.76 – 76.98***	15-Nov	16-May
Food groups										
Vegetables (g/d)	10.17	4.90	-3.41 – 23.76	19-Jul	19-Jan	5.19	2.46	-2.71 – 13.09	27-Sep	28-Mar
Fruit (g/d)	3.05	1.09	-20.92 – 27.02	0-Jan	1-Jul	3.05	1.06	-10.41 – 16.51	10-Dec	10-Jun
Whole grain products (g/d)	1.45	1.06	-6.85 – 9.74	21-Apr	20-Oct	3.77	3.09	**0.14 – 7.39***	10-Feb	10-Aug
Legumes (g/d)	2.76	32.66	**0.75 – 4.77***	7-Jan	7-Jul	3.79	40.80	**2.78 – 4.80***	29-Dec	29-Jun
Nuts (g/d)	0.95	10.90	-0.47 – 2.37	14-Apr	14-Oct	1.01	12.43	**0.30 – 1.71***	7-Jan	8-Jul
Dairy (g/d)	21.79	5.84	-5.04 – 48.63	16-Jul	16-Jan	17.99	4.95	**4.63 – 31.35***	10-Jun	10-Dec
Fish (g/d)	0.24	1.82	-1.34 – 1.83	1-Dec	1-Jun	2.04	13.35	**1.00 – 3.07***	30-May	29-Nov
Tea (mL/d)	17.03	5.30	-11.59 – 45.64	1-Apr	30-Sep	25.02	8.97	**11.45 – 38.59***	2-Feb	3-Aug
Unsaturated fats and oils ratio	1.07	1.54	-1.63 – 3.76	4-Nov	5-May	0.30	0.45	-1.11 – 1.71	20-Sep	21-Mar
Whole grains ratio	2.20	4.21	-1.08 – 5.48	25-May	23-Nov	0.77	1.47	-0.87 – 2.41	4-Jan	4-Jul
Red and processed meat (g/d)	2.93	3.58	-1.64 – 7.51	29-Sep	31-Mar	2.45	2.66	-0.23 – 5.12	10-Nov	11-May
Sugar-containing beverages (mL/d)	11.80	16.19	-1.15 – 24.74	1-Jun	1-Dec	13.29	17.44	**6.79 – 19.80***	31-May	30-Nov
Alcohol (g/d)	0.45	4.33	-0.82 – 1.72	8-Jan	9-Jul	0.69	5.85	-0.07 – 1.45	23-Jun	22-Dec
Salt (mg/d)	53.71	0.96	-79.75 – 187.18	21-Jan	21-Jul	88.51	1.56	**11.80 – 165.22***	11-Feb	11-Aug

Seasonal variation adjusted for cosinor terms, age, sex, cohort, physical activity, smoking behaviour, body mass index, comorbidities, education, and kilocalories/day (except when used as outcome).

** Seasonal variation expresses the maximum difference between the average highest intake/score (peak) and average lowest intake/score (nadir)

* Seasonal variation is statistically significant

[1] (Seasonal variation/average intake or score)*100%

Online Supplementary Material

Supplemental Table 3d. Stratified analyses: Seasonal variation of diet quality score and of each contributing food group according to socioeconomic status

	Lower SES n=8,937 observations					Higher SES n=3,652 observations				
**Outcome**	**Seasonal variation****	**%[1]**	**95%-confidence interval**	**Peak**	**Nadir**	**Seasonal variation****	**%[1]**	**95%-confidence interval**	**Peak**	**Nadir**
Diet quality score (0-14)	0.09	1.34	-0.01 – 0.20	3-Jan	4-Jul	0.13	1.94	-0.03 – 0.29	5-Dec	5-Jun
Kilocalories/day	42.62	2.12	**10.51 – 74.73***	4-Dec	5-Jun	57.81	2.62	**3.31 – 112.30***	20-Nov	22-May
Food groups										
Vegetables (g/d)	2.26	1.11	-5.29 – 9.81	13-Jul	12-Jan	12.69	5.62	-1.08 – 26.46	24-Sep	26-Mar
Fruit (g/d)	10.85	3.91	-2.24 – 23.95	16-Dec	16-Jun	15.47	5.11	-8.60 – 39.55	26-Jun	25-Dec
Whole grain products (g/d)	3.10	2.54	-0.89 – 7.09	5-Mar	4-Sep	3.90	2.92	-2.19 – 9.98	29-Dec	30-Jun
Legumes (g/d)	2.66	33.09	**1.64 – 3.69***	5-Jan	6-Jul	5.77	49.46	**3.95 – 7.59***	24-Dec	24-Jun
Nuts (g/d)	0.66	9.15	-0.05 – 1.37	21-Jan	22-Jul	1.05	9.74	-0.21 – 2.31	2-Feb	2-Aug
Dairy (g/d)	13.70	3.73	-0.81 – 28.21	2-Jun	2-Dec	30.23	8.36	**9.05 – 51.41***	29-Jun	29-Dec
Fish (g/d)	1.17	8.65	**0.17 – 2.17***	9-May	8-Nov	2.79	15.52	**1.00 – 4.57***	26-Jun	26-Dec
Tea (mL/d)	20.15	6.66	**5.31 – 34.98***	7-Mar	5-Sep	32.68	12.89	**10.91 – 54.45***	1-Jan	2-Jul
Unsaturated fats and oils ratio	0.63	0.92	-0.84 – 2.10	28-Aug	27-Feb	1.13	1.63	-1.00 – 3.26	2-Dec	2-Jun
Whole grains ratio	0.76	1.47	-1.02 – 2.53	6-Jan	7-Jul	1.14	2.10	-1.45 – 3.73	23-May	22-Nov
Red and processed meat (g/d)	2.48	2.77	-0.26 – 5.22	25-Oct	26-Apr	2.21	2.46	-2.14 – 6.56	17-Nov	19-May
Sugar-containing beverages (mL/d)	12.69	18.24	**5.82 – 19.56***	16-May	14-Nov	18.73	20.80	**7.81 – 29.66***	7-Jul	7-Jan
Alcohol (g/d)	0.46	4.64	-0.27 – 1.20	18-Jun	17-Dec	0.24	1.57	-1.09 – 1.57	15-Jul	15-Jan
Salt (mg/d)	78.70	1.43	**1.74 – 155.66***	13-Mar	12-Sep	171.33	2.86	**36.45 – 306.20***	27-Dec	28-Jun

SES = Socioeconomic status

Seasonal variation adjusted for cosinor terms, age, sex, cohort, physical activity, smoking behaviour, body mass index, comorbidities, education, and kilocalories/day (except when used as outcome).

** Seasonal variation expresses the maximum difference between the average highest intake/score (peak) and average lowest intake/score (nadir)

* Seasonal variation is statistically significant

[1] (Seasonal variation/average intake or score)*100%

Online Supplementary Material

Supplemental Table 3e. Stratified analyses: Seasonal variation of diet quality score and of each contributing food group according to living status

	Living alone n=3,361 observations					Living with partner/relatives/others n=9,228 observations				
**Outcome**	**Seasonal variation****	**%1**	**95%-confidence interval**	**Peak**	**Nadir**	**Seasonal variation****	**%1**	**95%-confidence interval**	**Peak**	**Nadir**
Diet quality score (0-14)	0.06	0.88	-0.11 – 0.23	27-Mar	26-Sep	0.16	2.40	**0.06 – 0.26***	13-Dec	14-Jun
Kilocalories/day	15.54	0.80	-39.92 – 71.00	2-Apr	2-Oct	64.42	3.05	**32.01 – 96.83***	24-Nov	26-May
Food groups										
Vegetables (g/d)	4.59	3.33	-9.49 – 18.68	15-Feb	16-Aug	8.15	3.79	**0.51 – 15.80***	1-Sep	3-Mar
Fruit (g/d)	21.31	7.18	-2.54 – 45.16	8-Dec	8-Jun	3.35	1.19	-10.04 – 16.75	16-Jun	16-Dec
Whole grain products (g/d)	3.94	3.31	-2.88 – 10.76	3-May	2-Nov	4.26	3.34	**0.45 – 8.06***	19-Jan	20-Jul
Legumes (g/d)	2.47	25.94	**0.49 – 4.46***	27-Dec	27-Jun	4.04	45.22	**3.06 – 5.02***	28-Dec	29-Jun
Nuts (g/d)	0.84	11.81	-0.42 – 2.11	1-Mar	31-Aug	0.88	10.16	**0.16 – 1.61***	16-Jan	17-Jul
Dairy (g/d)	21.43	5.49	-4.51 – 47.37	10-Jul	9-Jan	14.47	4.06	**1.17 – 27.77***	12-Jun	12-Dec
Fish (g/d)	0.47	3.08	-1.50 – 2.44	2-May	1-Nov	1.79	12.22	**0.81 – 2.76***	1-Jun	1-Dec
Tea (mL/d)	3.03	1.02	-22.84 – 28.89	20-Sep	22-Mar	27.47	6.63	**13.56 – 41.38***	10-Feb	10-Aug
Unsaturated fats and oils ratio	0.26	0.38	-2.23 – 2.75	17-Dec	18-Jun	0.62	0.91	-0.81 – 2.05	20-Oct	20-Apr
Whole grains ratio	3.08	6.31	-0.04 – 6.19	1-May	31-Oct	1.10	2.05	-0.60 – 2.79	3-Dec	3-Jun
Red and processed meat (g/d)	4.04	5.03	-0.24 – 8.31	30-Nov	31-May	1.98	2.13	-0.78 – 4.74	7-Oct	7-Apr
Sugar-containing beverages (mL/d)	12.63	17.93	**1.43 – 23.83***	20-Apr	20-Oct	14.80	19.13	**8.04 – 21.57***	12-Jun	11-Dec
Alcohol (g/d)	0.68	7.77	-0.45 – 1.82	14-Sep	15-Mar	0.54	4.33	-0.27 – 1.35	2-Jun	2-Dec
Salt (mg/d)	120.89	2.26	-8.21 – 249.99	24-Apr	23-Oct	111.36	1.93	**33.74 – 188.97***	9-Jan	10-Jul

Seasonal variation adjusted for cosinor terms, age, sex, cohort, physical activity, smoking behaviour, body mass index, comorbidities, education, and kilocalories/day (except when used as outcome).

** Seasonal variation expresses the maximum difference between the average highest intake/score (peak) and average lowest intake/score (nadir)

* Seasonal variation is statistically significant

1 (Seasonal variation/average intake or score)*100%

Online Supplementary Material

Supplemental Table 4. Stratified analyses: Seasonal variation of diet quality according to FFQ

Outcome	Seasonal variation*	95%-confidence interval	Peak	Nadir
**FFQ 170 items, n= 8,572.observations**				
Diet quality score (0-14)	0.10	-0.01 - 0.20	2-Jan	3-Jul
**FFQ 389 items, n= 4,017 observations**				
Diet quality score (0-14)	0.13	-0.02 - 0.29	17-Dec	18-Jun

FFQ = Food Frequency Questionnaire.

Seasonal variation adjusted for cosinor terms, age, sex, cohort, physical activity, smoking behaviour, body mass index, comorbidities, education, and kilocalories/day.

** Seasonal variation expresses the maximum difference between the average highest intake/score (peak) and average lowest intake/score (nadir)

Online Supplementary Material

Supplemental Table 5. Characteristics of the study population stratified by participants with a lower, intermediate, and higher diet quality score

	Diet score below 1SD of average* (<6.145) n=2,071		Diet score between -1 SD and + 1 SD of average* n=8,549		Diet score above 1SD of average* (>7.302) n=1,969	
**Covariate**	**Median (IQR)**		**Median (IQR)**		**Median (IQR)**	
Age (years)	65.9 (59.7 - 71.9)		66.8 (59.8 - 73.9)		63.4 (57.3 - 75.12)	
Physical activity (MET-hours per week)	43.0 (21 - 70.5)		62.4 (30.3 - 95.1)		85.3 (47.4 – 128.0)	
BMI, kg/m^2	27.0 (24.7 - 29.6)		26.6 (24.4 - 29.3)		25.2 (23.2 - 27.6)	
Kilocalories/day	1942 (1589 – 2312)		1946 (1612 – 2336)		2264 (1934 – 2721)	
	**N**	**%**	**N**	**%**	**N**	**%**
Sex						
men	1,767	85.3	3,414	39.9	125	6.4
women	304	14.7	5,135	60.1	1,844	93.6
Education						
primary	276	13.4	1,227	14.5	192	9.8
lower	854	41.5	3,640	43.0	695	35.5
intermediate	748	36.4	2,363	27.9	515	26.4
higher	178	8.7	1,236	14.6	554	28.3
Smoking status						
never	84	4.0	2,671	31.4	1,276	65.2
ever	626	30.3	4,594	54.0	659	98.7
current	1,359	65.7	1,246	14.6	21	1.1
Comorbidities						
no	1,566	75.6	7,089	82.9	1,779	90.4
yes	505	24.4	1,460	17.1	190	9.6
Socioeconomic status						
lower	1,438	69.4	6,213	72.7	1,286	65.3
higher	633	30.6	2,336	27.3	683	34.7
Living status						
alone	389	18.8	2,285	26.7	687	34.9
with others	1,682	81.2	6,264	73.3	1,282	65.1

BMI = Body mass index. IQR = Interquartile range. MET = Metabolic Equivalent of Task. SD = Standard deviation.

*Predicted average of diet score after adjusted for covariates of Model 2.
